# Long-term efficacy and safety of a treatment strategy for HIV infection using protease inhibitor monotherapy: 8-year routine clinical care follow-up from a randomised, controlled, open-label pragmatic trial (PIVOT)

**DOI:** 10.1016/j.eclinm.2024.102457

**Published:** 2024-02-10

**Authors:** Nicholas I. Paton, Wolfgang Stöhr, Alejandro Arenas-Pinto, Amanda Clarke, Ian Williams, Margaret Johnson, Chloe Orkin, Fabian Chen, Vincent Lee, Alan Winston, Mark Gompels, Julie Fox, Karen Sanders, David T. Dunn, Protease Inhibitor monotherapy Versus Ongoing Triple therapy (PIVOT) Trial Team, Martin Fisher, Amanda Clarke, Wendy Hadley, David Stacey, Margaret Johnson, Pat Byrne, Ian Williams, Nahum De Esteban, Pierre Pellegrino, Lewis Haddow, Alejandro Arenas-Pinto, Chloe Orkin, James Hand, Carl De Souza, Lisa Murthen, Andrew Crawford-Jones, Fabian Chen, Ruth Wilson, Elizabeth Green, John Masterson, Vincent Lee, Kamlesh Patel, Rebecca Howe, Alan Winston, Scott Mullaney, Mark Gompels, Louise Jennings, Nicholas Beeching, Rebecca Tamaklo, Julie Fox, Alistair Teague, Isabelle Jendrulek, Juan Manuel Tiraboschi, Ed Wilkins, Yvonne Clowes, Andrew Thompson, Gary Brook, Manoj Trivedi, Kazeem Aderogba, Martin Jones, Andrew DeBurgh-Thomas, Liz Jones, Iain Reeves, Sifiso Mguni, David Chadwick, Pauline Spence, Nellie Nkhoma, Zoe Warwick, Suzanne Price, Sally Read, Elbushra Herieka, James Walker, Ruth Woodward, John Day, Laura Hilton, Veerakathy Harinda, Helen Blackman, Phillip Hay, Wendy Mejewska, Olanike Okolo, Edmund Ong, Karen Martin, Lee Munro, David Dockrell, Lynne Smart, Jonathan Ainsworth, Anele Waters, Stephen Kegg, Sara McNamara, Steve Taylor, Gerry Gilleran, Brian Gazzard, Jane Rowlands, Sris Allan, Rumun Sandhu, Nigel O'Farrell, Sheena Quaid, Fabiola Martin, Caroline Bennett, Moses Kapembwa, Jane Minton, James Calderwood, Frank Post, Lucy Campbell, Emily Wandolo, Adrian Palfreeman, Linda Mashonganyika, Thambiah Balachandran, Memory Kakowa, Rebecca O'Connell, Cheryl Tanawa, Sinna Jebakumar, Lesley Hagger, Say Quah, Sinead McKernan, Charles Lacey, Sarah Douglas, Sarah Russell-Sharpe, Christine Brewer, Clifford Leen, Sheila Morris, Sharmin Obeyesekera, Shirley Williams, Nelson David, Mark Roberts, Julie Wollaston, Nicholas Paton, Wolfgang Stöhr, Alejandro Arenas-Pinto, Karen Scott, David Dunn, Emma Beaumont, Sue Fleck, Mark Hall, Susie Hennings, Ischa Kummeling, Sara Martins, Ellen Owen-Powell, Karen Sanders, Fionna van Hooff, Livia Vivas, Ellen White, Brian Angus, Andrew Freedman, Ben Cromerty, Danielle Mercey, Sarah Fidler, Estee Torok, Abdel Babiker, Brian Gazzard, Chloe Orkin, Nicholas Paton, Tim Peto, David Lalloo, Andrew Phillips, Robert James

**Affiliations:** a MRC Clinical Trials Unit at University College London, London, UK; b Yong Loo Lin School of Medicine, National University of Singapore, Singapore; c University Hospitals Sussex NHS Foundation Trust, Brighton, UK; d University College London, UK; e Royal Free Hospital, London, UK; f Barts and the Royal London Hospital NHS Trust, London, UK; g Royal Berkshire Hospital, Reading, UK; h Manchester Royal Infirmary, UK; i St Mary's Hospital, London, UK; j Southmead Hospital, Bristol, UK; k Guys and St. Thomas' Hospital, London, UK

**Keywords:** HIV, Randomised, Protease inhibitor monotherapy, Simplification, Darunavir

## Abstract

**Background:**

Treatment-simplification strategies are important tools for patient-centred management. We evaluated long-term outcomes from a PI monotherapy switch strategy.

**Methods:**

Eligible participants attending 43 UK treatment centres had a viral load (VL) below 50 copies/ml for at least 24 weeks on combination ART. Participants were randomised to maintain ongoing triple therapy (OT) or switch to a strategy of physician-selected PI monotherapy (PI-mono) with prompt return to combination therapy if VL rebounded. The primary outcome, previously reported, was loss of future drug options after 3 years, defined as new intermediate/high level resistance to at least one drug to which the participant's virus was considered sensitive at trial entry. Here we report resistance and disease outcomes after further extended follow-up in routine care. The study was registered as ISRCTN04857074.

**Findings:**

We randomised 587 participants to OT (291) or PI-mono (296) between Nov 4, 2008, and July 28, 2010 and followed them for a median of more than 8 years (100 months) until 2018. At the end of this follow-up time, one or more future drug options had been lost in 7 participants in the OT group and 6 in the PI-mono group; estimated cumulative risk by 8 years of 2.7% and 2.1% respectively (difference −0.6%, 95% CI −3.2% to 2.0%). Only one PI-mono participant developed resistance to the protease inhibitor they were taking (atazanavir). Serious clinical events (death, serious AIDS, and serious non-AIDS) were infrequent; reported in a total of 12 (4.1%) participants in the OT group and 23 (7.8%) in the PI-mono group (P = 0.08) over the entire follow-up period.

**Interpretation:**

A strategy of PI monotherapy, with regular VL monitoring and prompt reintroduction of combination treatment following rebound, preserved future treatment options. Findings confirm the high genetic barrier to resistance of the PI drug class that makes them well suited for creative, patient-centred, treatment-simplification approaches. The possibility of a small excess risk of serious clinical events with the PI monotherapy strategy cannot be excluded.

**Funding:**

The National Institute for Health Research Health Technology Assessment programme.

**Research in context**

**Evidence before this study**

We searched PubMed for reports published up to June 30, 2023 that described randomised controlled trials comparing PI monotherapy with triple antiretroviral therapy (ART) in patients with viral load suppression. A meta-analysis, published in 2016, of 13 randomised controlled trials comparing PI monotherapy with triple ART, showed that viral load suppression was significantly lower with PI monotherapy when treatment switches were counted as failures but not when they were ignored in an intention-to-treat analysis. The longest reported follow-up in any of the component trials in that meta-analysis was 44 months (from this trial, when reporting the primary outcome).

**Added value of this study**

The extended follow-up allows meaningful assessment of the long-term consequences arising from episodes of viral load rebound, experienced by a high proportion of patients, mostly in the first year following switch to PI monotherapy. The new data show no difference between PI-mono and OT groups on the main outcome–loss of future treatment options–with just one case of PI drug resistance during the entire period (previously reported); no detrimental impact of PI monotherapy on viral non-suppression after the first year of follow-up; but a greater number of serious clinical events in the PI monotherapy group (not statistically significant). At the end of follow-up, over 30% of participants remained on PI monotherapy.

**Implications of all the available evidence**

A strategy of switching stable, virologically-suppressed patients to PI monotherapy with close monitoring and re-institution of combination therapy in those who rebound, preserves future drug options and long-term viral suppression but the possibility of a small excess risk of serious clinical events cannot be excluded. The strategy appears to retain acceptability amongst patients and their clinicians within a system that permits individualised, patient-centred care.

## Introduction

Although use of three-drug regimens was initially considered essential for effective antiretroviral therapy, it is now broadly accepted that once viral suppression has been attained it is possible to switch to regimens with fewer drugs and sustain suppression; with possible advantages including reduced toxicity and cost.

Protease inhibitors (PIs) have a high genetic barrier to resistance and were used as the foundation of regimens in the first studies of such treatment simplification approaches. Multiple randomised controlled trials comparing PI monotherapy to continued triple therapy demonstrated that high rates of suppression were maintained,^1^, ^2^, ^3^, ^4^, ^5^ with a minority of patients having viral load rebound necessitating re-introduction of nucleoside reverse transcriptase inhibitors (NRTIs). A meta-analysis showed that PI monotherapy was inferior to triple therapy in a conventional regimen efficacy analysis, where switch (in this case addition of NRTIs) counts as failure.^6^ Subsequent studies showed that combining a PI with lamivudine abolished the excess risk of viral load rebound seen with PI monotherapy, achieving rates of viral suppression that are non-inferior to triple therapy; and this has subsequently been extended to a dual combination with dolutegravir (a second-generation integrase inhibitor, also with a high genetic barrier to resistance) and lamivudine.^7^ These and other dual therapy regimens have shown non-inferiority to standard triple therapy when used as a treatment switch option after viral suppression has been attained; have been recommended in treatment guidelines^8^; and are becoming widely used in clinical practice.

The Protease Inhibitor monotherapy Versus Ongoing Triple therapy (PIVOT) trial was the largest of the trials that investigated PI monotherapy and had the longest follow-up.^9^ It differed from other trials in evaluating a specified PI monotherapy *strategy*, which included close viral load monitoring and prompt re-introduction of NRTIs when viral load rebound was confirmed, designed to reflect the way such an intervention would be used in clinical practice. The primary endpoint was loss of future drug options (due to development of viral resistance) rather than the conventional endpoint of failure of viral suppression. This design and endpoint were chosen to be most relevant for assessing the long-term consequences of the PI monotherapy strategy, balancing the competing risks of the expected higher rate of viral rebound with PI monotherapy against the possible lower risk of resistance development when treatment comprises only a drug from a class with a known high genetic barrier to resistance. The main trial results, after a median of 44 months of follow-up, demonstrated that PI monotherapy was non-inferior to triple therapy on this outcome.^9^

Upon completion of the protocol-directed management stage of the trial, we continued to collect relevant outcome data acquired through routine clinical care delivered at the trial sites. The specific objectives of this were to describe retention on PI monotherapy, risks of late viral rebound and drug resistance, and safety of the PI monotherapy strategy versus standard combination therapy over long-term follow-up; and overall to strengthen the long-term evidence base of randomised controlled data to support rational decision-making on the risks and benefits of treatment simplification options using PIs in clinical practice.

## Methods

### Study design and participants

PIVOT was a pragmatic, parallel-group, randomised, controlled, open-label non-inferiority trial that was conducted in two stages. In the initial, protocol-directed management stage, treatment switches, viral load and safety monitoring were performed according to the schedule and algorithms specified in the trial protocol. Participants who were willing then continued in the trial in a second, routine clinical management stage during which treatment and monitoring were at the discretion of clinician.

Participants were enrolled at 43 public sector hospital-based HIV treatment centres in the UK. The main inclusion criteria were being HIV positive, aged ≥18 years, taking ART consisting of two nucleoside reverse transcriptase inhibitors (NRTIs) and one non-NRTI (NNRTI) or protease inhibitor for at least 24 weeks, a viral load of <50 copies per mL at screening and for at least 24 weeks before screening, and a CD4 count >100 cells per μL at screening. The main exclusion criteria were known major protease inhibitor resistance mutations on any previous resistance test and previous ART change for unsatisfactory virological response. Detailed eligibility criteria have been reported previously.^9^

### Ethics statement

The original protocol and amendment to include the second stage was approved by the Cambridgeshire 4 Research Ethics Committee and Medicines and Healthcare Products Regulatory Agency. All participants provided written informed consent for the initial stage of the trial; separate written informed consent was obtained from those participants willing to continue to the second stage of the trial.

### Randomisation and masking

Participants were randomly assigned (1:1) to maintain ongoing triple therapy (OT) or switch to a protease inhibitor monotherapy strategy (PI-mono). Randomisation was stratified by centre and baseline ART regimen and was performed at the coordinating centre.^9^

### Procedures

In the protocol-directed management stage, participants randomised to the OT group were managed using standard triple therapy, with the choice of regimen at discretion of clinician and participant. Participants randomised to the PI-mono group were switched to a single ritonavir-boosted protease inhibitor selected by the physician (the protocol recommended ritonavir-boosted darunavir or lopinavir). Protease inhibitor substitution was allowed in the event of toxicity or for convenience. The strategy required prompt reintroduction of NRTIs for protocol-defined confirmed viral load rebound and management with combination treatment thereafter. Viral load was measured every 12 weeks in both groups; genotypic resistance testing was done on all viral load rebound samples that were confirmed or preceded treatment switch. The treatment and monitoring strategy has been described in detail.^9^ This stage ended on 1 November 2013, after a median follow-up duration of 44 (maximum 59) months. In the subsequent routine clinical management stage, continued until 2018, treatment and monitoring were at the discretion of clinician and participant, following routine clinical care and without any protocol-mandated requirements for monitoring tests.

In the initial stage, detailed outcomes on clinical, laboratory and participant self-reported variables were collected on case report forms completed for every protocol-mandated trial visit, as previously described.^9^ In the second stage, data on a reduced set of core variables were collected on case report forms for each participant (Appendix p 6) that were distributed by the coordinating centre for completion during the first quarter of 2015, 2016, and 2018. The forms collected data on all local clinical care received following the last case report that had been submitted. The form elicited information on date of last clinical visit, whether the participant was still under follow-up, the ART regimen(s) taken since the previous data collection and reasons for any changes, the results and dates of all viral load and resistance tests, the results and dates of the most recent CD4 count and serum creatinine measurements, and details of any serious AIDS-defining illnesses (excluding oesophageal candidiasis or chronic mucocutaneous herpes simplex virus infection) or serious non-AIDS events.

### Outcomes

The primary outcome of the trial was loss of future drug options, defined as new intermediate-level or high-level resistance to one or more drugs in contemporary use (defined by successive British HIV Association treatment guidelines, that were in current use at any time point during the trial follow-up period) to which we deemed the participant's virus to be sensitive at trial entry (assessed at 3 years of follow-up).^9^ The main outcome for the analysis presented here is the same, but included all mutations observed until the end of follow-up. A pre-specified sensitivity analysis restricted the loss of future drug options to classes to which the participant was exposed during the trial (before detection of drug resistance), with other mutations considered to be probably archived.

Other outcomes for this analysis were viral failure (defined as a single viral load result ≥200 copies/ml, applied during the entire trial follow-up; this differed from the definition used for the initial stage because sites did not necessarily follow the earlier protocol-mandated testing strategy during the second stage); viral non-suppression (≥200 copies/ml) at annual time points from randomisation; change in CD4 count and change in serum creatinine from baseline to last available measurement; and the proportion of participants who died or experienced a new serious AIDS event or serious non-AIDS event.

### Statistical analysis

For this report, data collected during both stages were combined in the analysis. Switch from allocated therapy was defined as discontinuation of all ART for more than 28 days (both groups), the re-introduction of combination therapy (PI-mono group), or the initiation of PI monotherapy (OT group). This outcome was analysed using time-to-event methods, censoring at the date of last clinic visit. The loss of future drug options was also analysed using time-to-event methods, censoring at the date of the last viral load measurement. A similar analysis was used for the first viral failure event except that censoring in PI-mono group also included switch from the allocated therapy (for any reason). This analysis therefore reflects the experience of those participants who stayed on their allocated PI monotherapy while remaining virologically suppressed.

Other outcomes were analysed in the intention-to-treat population. Proportions (viral load suppression at specified time-points, death, serious AIDS and non-AIDS events) were compared between groups using Fisher's exact tests, with Agresti-Caffo 95% CIs for the risk difference. Change in CD4 count and creatinine were compared between groups by t-tests, after checking for approximate normality. Data were analysed “as observed” i.e. assuming that data were missing at random. The basis for the estimation of sample size has been described previously.^9^ Analyses for this report used Stata version 15. The trial is registered with the International Standard Randomised Controlled Trial Number registry, number ISRCTN04857074.

### Role of the funding source

The trial was funded by the National Institute for Health Research Health Technology Assessment programme. The funder of the study had no role in study design, data collection, data analysis, data interpretation, or writing of the report or the decision to submit for publication. WS and DD accessed the dataset and NP had final responsibility for the decision to submit for publication.

## Results

Between Nov 4, 2008, and July 28, 2010 we randomised 587 participants to OT (291) or PI-mono (296) from 43 sites in the UK. Of these, 23 participants (12 OT, 11 PI-mono) died, withdrew, or were lost to follow-up before the end of the first stage of the trial (Appendix p 5). Of the 564 participants that completed the first stage, 505 (90%) consented to take part in the routine clinical follow-up stage (Appendix, p 5). The baseline characteristics of participants enrolled in the first and second stages of the trial were similar (Table 1; Appendix p 4). Overall, 95% of expected forms were returned (Appendix, p 3). The median follow-up from randomisation to last clinic visit was 100 months (maximum 118 months), compared with 44 months (59 months) at the end of the first stage of the trial.^9^

**Table 1:** Baseline characteristics at randomisation, all participants and those with additional follow-up.

	All participants (n = 587)	Participants with additional follow-up (n = 505)
Trial group		
OT	291 (50%)	257 (51%)
PI-mono	296 (50%)	248 (49%)
Drug class at entry		
PI	273 (47%)	233 (46%)
NNRTI	314 (53%)	272 (54%)
Age (years)	44 (38–49)	44 (38–49)
Female sex^a^	137 (23%)	118 (23%)
Route of infection		
Homosexual	351 (60%)	304 (60%)
Heterosexual	216 (37%)	181 (36%)
Other	20 (3%)	20 (4%)
Ethnicity		
White	401 (68%)	349 (69%)
Black	163 (28%)	133 (26%)
Other	23 (4%)	23 (5%)
Nadir CD4 count (cells per mm^3^)	178 (86–250)	180 (86–250)
Baseline CD4 count (cells per mm^3^)	513 (392–682)	514 (393–676)
Duration undetectable VL (months)	37 (20–63)	38 (20–65)

Data are n (%) or median (IQR). OT = ongoing triple therapy. PI-mono = protease inhibitor monotherapy. NNRTI = non-nucleoside reverse transcriptase inhibitor.

a Female sex was sex assigned at birth.

**Fig. 1:**
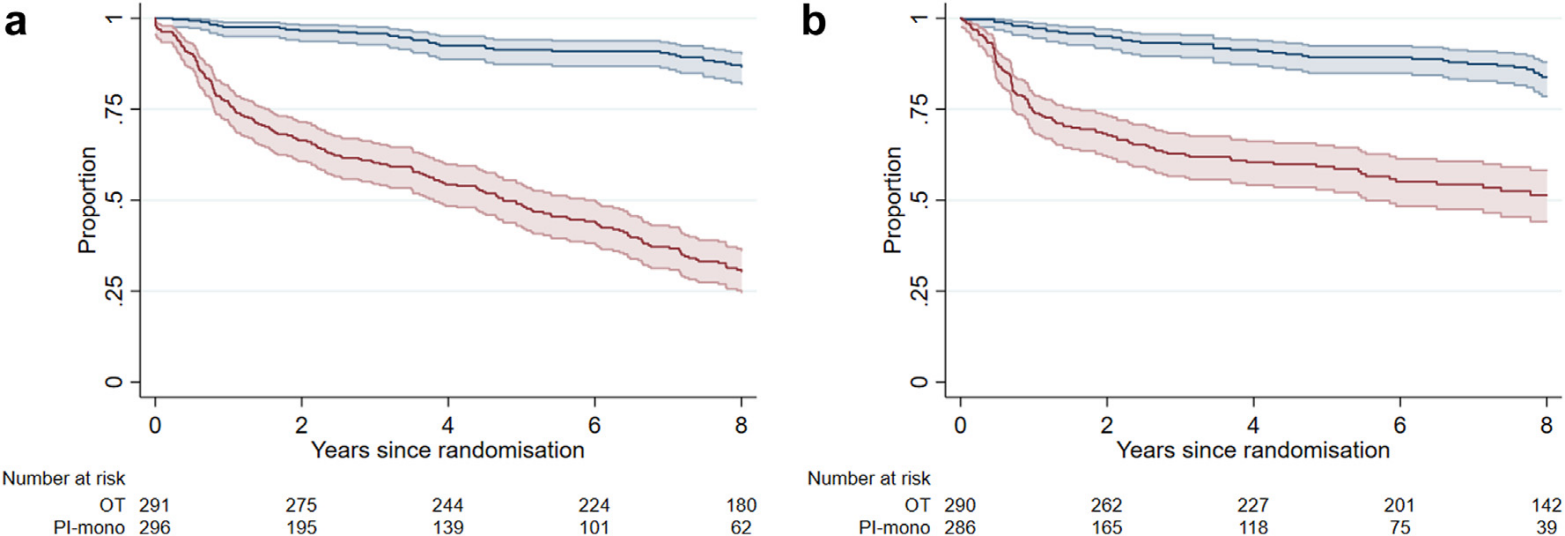
a). **Kaplan–Meier estimates of the proportion of participants who remained on the treatment allocated by randomisation**. The ongoing triple therapy (OT) group is shown in blue; the protease inhibitor monotherapy (PI-mono) group is shown in red. Switch from allocated treatment was defined as discontinuation of all ART for ≥28 days (both groups); or re-introduction of combination therapy (PI-mono group); or initiation of PI monotherapy (OT group). Follow-up was censored at the last clinic visit. b). **Kaplan–Meier estimates of the proportion of participants who remained on the treatment allocated by randomization who did not experience viral failure**. The ongoing triple therapy (OT) group is shown in blue; the protease inhibitor monotherapy (PI-mono) group is shown in red. Viral failure was defined as a single VL ≥ 200 copies/ml. Follow-up was censored at switch from allocated treatment (defined as above) or at last viral load measurement.

In the PI-mono group, initial drug choices were darunavir in 233 (80%) participants, increasing during follow-up to 250 (84%) with switches from other protease inhibitors (for convenience or tolerability, while virologically suppressed); lopinavir in 40 (14%); atazanavir in 16 (6%); saquinavir in 1 (<1%); and 6 (2%) never started monotherapy. Protease inhibitors were boosted with low-dose ritonavir or cobicistat. An estimated 23.4% of participants discontinued PI monotherapy during the first year, declining to 13.0% per year in subsequent years; 48.9% remained on PI monotherapy at 5 years, and 30.3% at 8 years from randomisation (Fig. 1a). The total follow-up time spent on monotherapy was 1246 participant-years. The reasons given for discontinuing PI monotherapy were viral rebound in 97 (51%); clinician or participant decision in 34 (18%); adverse effects in 28 (15%); and other or unknown reason in 32 (17%). There was no evidence (log rank P = 0.52) that the rate of discontinuation varied by the specific PI monotherapy drug (Appendix p 6). At discontinuation, 173 participants switched to triple therapy (the majority retaining the PI and adding NRTIs); 16 to dual therapy (adding lamivudine or emtricitabine in 14, raltegravir in 1, dolutegravir in 1); and 2 had no record of re-starting other therapy during the remainder of trial follow-up.

In the OT group, an estimated 86.6% remained on combination therapy at 8 years from randomisation (Fig. 1a), although many had changed from their baseline combination. The last recorded regimen, at the end of follow-up, included an NNRTI in 43.6%, a PI in 33.3%, and an integrase inhibitor in 24.4%.

By the end of the trial follow-up, 7 participants in the OT group (4 in the first stage, 3 in the second stage) and 6 participants in the PI-mono group (all in the first stage) met the main outcome definition of loss of future drug options (Table 2). The estimated cumulative risk of experiencing this outcome by 8 years was 2.7% in the OT group compared with 2.1% in the PI-mono group (difference −0.6%, 95% CI −3.2% to 2.0%). In the pre-specified sensitivity analysis excluding mutations that were probably archived, 6 participants in the OT group and 3 participants in the PI-mono group met this outcome definition with an estimated cumulative risk by 8 years of 2.3% and 1.1% respectively (difference −1.3%, 95% CI −3.5% to 1.0%). During the entire follow-up period, only one participant in the PI-mono group developed a clinically significant protease mutation to the drug they were taking (I50L whilst on atazanavir; reported during the first stage).

**Table 2:** Primary outcome—loss of future drug options.

Participant	Drugs received during trial before date of resistance test	Reverse Transcriptase mutations	Protease mutations	Lost drug options
OT group				
1	ABC, 3TC, ATV	V118I, V179D, M184V	I84V	3TC, FTC, SQV, FPV, TPV
2	TDF, FTC, RPV, DRV	L100I, K103N, M184V	A71V	3TC, FTC, NVP, EFV, ETV, RPV
3	TDF, FTC, ETV, NVP, EFV	M184V/I, K65R, E138A, Y181C, H221Y, M230L	–	3TC, FTC, ABC, TDF, NVP, EFV, ETV, RPV
4	TDF, FTC, DRV	V106A	–	NVP^b^, EFV^b^
5	ZDV, 3TC, TDF, FTC, NVP, DRV	V179D, M184LV	–	3TC
6	DDI, 3TC, ABC, EFV, DRV	K103N	–	NVP, EFV
7	ZDV, TDF, 3TC, FTC, EFV	V106M, Y188HY	–	NVP, EFV, DOR
PI-mono group				
1	ATV	–	K20T, I50L/I, A71T	ATV
2	DRV	–	L90M	SQV^a^
3	DRV	–	A71T, L90M	SQV^a^
4	DRV	K103N	–	NVP^b^, EFV^b^
5	DRV	K103N	–	NVP^b^, EFV^b^
6	DRV	M41L, T215D	–	ZDV^b^

Loss of future drug options is defined as new intermediate/high level resistance to one or more drugs in contemporary use to which the participant's virus was considered to be sensitive at trial entry. Table shows individual participants meeting the main endpoint. ABC, abacavir; 3TC, lamivudine; FTC, emtricitabine; TDF, tenofovir; ZDV, zidovudine; SQV, saquinavir; FPV, fosamprenavir; TPV, tipranavir; ATV, atazanavir; NVP, nevirapine; EFV, efavirenz; ETV, etravirine; RPV, rilpivirine; DOR, doravirine.

a Possibly archived resistance (mutations to drugs in classes to which the participant was exposed during the trial, but that are not selected by the specific drug to which the participant was exposed).

b Probably archived resistance (mutations to drugs in classes to which the participant was not exposed during the trial and were therefore likely present prior to enrolment).

Viral load testing was performed 12-weekly in the first stage of the trial with additional tests at 4 and 8 weeks in the PI-mono group (protocol-mandated); and at a median interval of 23 weeks (OT group) and 24 weeks (PI-mono group) in the second stage. The rate of viral failure in the PI-mono group was much higher in the first year (28.9 per 100 person-years, 95% CI 22.9–36.6) than in subsequent years (5.9 per 100 person-years, 95% CI 4.4–7.8); the rate in the OT group was similar throughout follow-up (2.2 per 100 person-years; overall rate ratio of PI-mono to OT: 4.4 [95% CI 3.1–6.2]; P < 0.001). Of the participants who remained on PI monotherapy (after censoring those who switched for reasons other than viral rebound), an estimated 59.3% (95% CI 52.9–65.1) and 51.4% (44.1–58.2) experienced no viral failure over 5 years and 8 years respectively (Fig. 1b). In a cross-sectional comparison, there was no systematic difference between the OT and PI-mono groups in the proportion with HIV viral load ≥200 copies/ml at yearly time points from randomisation, other than at the end of the first year (Table 3). The rate of non-suppression in the PI-mono group was typically between 1% and 3%.

**Table 3:** Proportion with HIV-1 RNA non-suppression (≥200 copies/ml) at annual time points from randomisation.

Years since randomisation	Number (%) with viral load ≥200 copies/ml		P-value
	OT	PI-mono	
1	2/287 (0.7)	16/295 (5.4)	<0.001
2	3/284 (1.1)	6/290 (2.1)	0.51
3	2/293 (0.7)	3/287 (1.1)	1.00
4	3/261 (1.2)	7/250 (2.8)	0.21
5	3/243 (1.2)	8/219 (3.6)	0.13
6	3/230 (1.3)	3/213 (1.4)	1.00
7	1/217 (0.5)	3/206 (1.5)	0.36
8	4/214 (1.9)	1/196 (0.5)	0.37

The mean increase in CD4 count from baseline to last available measurement was similar in the PI-mono group (143 [SE 12.6] cells/mm^3^) and the OT group (146 [SE 11.8]); the mean decrease in serum creatinine was marginally lower (P = 0.05) in the PI-mono group (9.08 [SE 0.86 μmol/L) compared with the OT group (11.41 [SE 0.81] μmol/L). There were an additional 2 participants who died (1 PI-mono, 1 OT), 3 who experienced a serious AIDS event (all PI-mono), and 10 who experienced a serious-non-AIDS event (6 PI-mono, 4 OT) during the second stage of the trial. Over the whole trial follow up, one or more of these serious clinical events were reported in a total of 12 (4.1%) participants in the OT group and 23 (7.8%) in the PI-mono group (P = 0.08; Table 4). Encephalopathy was reported in 3 participants, all taking darunavir monotherapy. One had memory impairment; the diagnosis was made clinically without additional investigations. One had transient visual impairment and slurred speech which resolved without change of treatment; MRI was consistent with HIV encephalopathy and virus was detected in the CSF. One was asymptomatic, with MRI changes detected on scan performed for another research study and virus detected in the CSF. None of the three had plasma viral load rebound.

**Table 4:** Serious clinical events related to HIV disease or treatment.

Outcome	OT (n = 291)	PI-mono (n = 296)	Difference (95% CI)	p-value
Total	12 (4.1%)	23 (7.8%)	3.6% (−0.3, 7.5)	0.08
Death^a^	2 (0.7%)	7 (2.4%)	1.7% (−0.5, 3.8)	0.18
Serious AIDS	1 (0.3%)	4 (1.4%)	1.0% (−0.7, 2.7)	0.37
Encephalopathy	0	3		
Extrapulmonary TB	0	1		
Cytomegalovirus colitis	1	0		
Serious non-AIDS	11 (3.8%)	17 (5.7%)	2.0% (−1.6, 5.5)	0.33
Cancer	7	14		
Cerebrovascular accident	1	0		
Facial wasting	1	0		
Myocardial infarction	1	1		
Renal failure	0	1		
Pancreatitis	1	1		

Data are n (%) of participants having the specified event during the entire trial follow-up period.

a Causes of death in the OT group were oesophageal adenocarcinoma and metastatic adenocarcinoma (unknown primary); and in the PI-mono group were suicide, pulmonary embolism, breast carcinoma (recurrent), small cell lung carcinoma, glioblastoma and anal carcinoma (2 cases).

## Discussion

Our finding of non-inferiority of the PI monotherapy strategy on the main outcome of loss of future drug options after 8 years from strategy initiation in each participant confirms the findings from the initial stage of the trial. Just one participant in the PI-mono group (taking atazanavir) developed a resistance mutation conferring intermediate-high level PI resistance. In contrast, six participants in the group randomised to continue triple therapy developed resistance to agents (NRTIs, NNRTIs, PIs) to which they were exposed during the trial. Although it is harder to differentiate these mutations with certainty from archived resistance, it is a plausible assumption that observed resistance to NRTIs and NNRTIs was generated during the documented episodes of rebound during the trial, given the known low genetic barrier to resistance in these drug classes.

The almost complete absence of PI resistance mutations during 1246 person-years follow-up on PI monotherapy, despite numerous, documented episodes of virological rebound, provides strong practical confirmation of the known high genetic barrier to resistance of this drug class (especially darunavir, which was the PI used as monotherapy in over 84% of the participants allocated to that strategy; and has the highest barrier to resistance within the class).^10^ Whilst it is possible that routine bulk sequencing done at clinical sites failed to detect mutations, a sub-study that performed highly-sensitive, next-generation sequencing during the initial stage of the trial found no additional, relevant, low-level resistant viral variants.^11^ Although second-generation drugs in the integrase inhibitor drug class, such as dolutegravir, are also considered to have a high genetic barrier to resistance, dolutegravir monotherapy trials were not successful: short-term rebound rates were relatively high and, in contrast to PI monotherapy, high rates of integrase resistance were seen.^12^^,^^13^ Furthermore, in the NADIA trial—which performed a head-to-head comparison of darunavir versus dolutegravir in second-line therapy on a backbone of NRTIs that, in most cases, had pre-existing resistance**—**9 participants (3.8%) developed new intermediate-high level dolutegravir resistance; in contrast, none on darunavir developed resistance.^14^^,^^15^ Taken together, the results of these studies, including PIVOT, that have challenged protease inhibitors and integrase inhibitors in paradigms with limited or no protection from NRTIs, demonstrate that protease inhibitors (in particular darunavir, but also lopinavir) are the most robust drug class in the HIV treatment armamentarium. However, PIs are not indestructible: progressive accumulation of resistance mutations was observed in a trial of lopinavir monotherapy used without real-time virological monitoring.^16^ This underscores the importance of prompt detection and reintroduction of NRTIs in patients with viral load rebound, which was an intrinsic part of the PIVOT strategy.

At the time this trial was designed, PI monotherapy was a popular treatment simplification approach used in a substantial minority of patients in many treatment centres in Europe, driven by concerns about long-term NRTI toxicity; the theoretical appeal of reserving NRTIs to maximise available options for later salvage regimens; and cost reduction with fewer drugs. However, each of these potential advantages has become less compelling over time. Newer NRTIs have reduced toxicity and our findings indicate no substantive long-term toxicity advantage from the PI monotherapy strategy; including on renal toxicity which was the main concern. It has also been conclusively demonstrated that activity of NRTIs is preserved despite resistance mutations when they are used in combination with a fully-active protease inhibitor or dolutegravir.^14^^,^^15^^,^^17^^,^^18^ The advent of second-generation integrase inhibitors with equivalent potency that can be manufactured well below the cost of PIs, have eliminated the cost-saving advantage. Furthermore, for those who need or wish to remain on a PI-based regimen, using dual therapy with atazanavir, lopinavir, or darunavir combined with the NRTI lamivudine, abolishes the excess risk of viral load rebound seen with PI monotherapy (discussed below), with minimal effect on toxicity and only marginal increase in cost^19^^,^^20^; and is recommended as a switch strategy in contemporary treatment guidelines.^8^^,^^21^^,^^22^

Potential disadvantages of the PI monotherapy strategy are those that might arise from the excess viral rebound. The rate of viral rebound was high in the first year after switch but decreased dramatically thereafter; and the median period of viral load suppression on PI monotherapy exceeded 8 years. Careful patient selection using factors shown to predict lower risk of viral load rebound (longer duration of viral load suppression before starting PI monotherapy and higher nadir CD4 cell count) could extend this further.^23^ Nevertheless, this complicates clinical management because of the need for careful viral load monitoring for early detection of rebound (required more frequently, at least initially, than in those who are stable on combination treatment); and because of the need for reintroduction of combination treatment in some people (although this is straightforward and re-suppression is rapid). The high rate of viral load rebound we observed in the first year, possibly reflecting reduced potency of PI monotherapy compared with triple therapy in some individuals, was deemed unacceptable in the context of traditional treatment guidelines criteria (although such criteria are not suited for evaluation of strategies, as discussed below) and contemporary guidelines explicitly rule out the use of PI monotherapy as a treatment regimen.^8^^,^^21^

This trial provides an opportunity to examine the risks of such viral rebound episodes on longer-term outcomes within the paradigm of a randomised controlled trial—there were over 160 observed rebound episodes (and possibly other transient rebound episodes that were unobserved between scheduled tests in the PI-mono group), a high proportion of which occurred early, and with a long period of subsequent follow-up. Historically, the disappointing results from trials of structured treatment interruptions (complete interruption of all ART), reinforced by the observation of new drug resistance mutations in those with active viral replication on low genetic barrier NRTI and NNRTI-based regimens, fomented the view that episodes of viral rebound are harmful; and that therapy should therefore aim to achieve continuous and complete viral suppression.^8^ In addition to finding no detrimental effect of viral rebound on future drug options (discussed above), we also found no substantive impact on the prospects for future viral suppression: after the first year the point prevalence of viral non-suppression was low (typically 1–3%), consistent with the rapid re-suppression that occurs following reintroduction of NRTIs for rebound.^9^^,^^23^ Other possible long-term adverse consequences of viral rebound include those mediated through pathways involving increased immune activation, that might affect immunological recovery or increase clinical events linked to inflammatory or immune responses.^24^^,^^25^ However, the short-term, low-level viral rebound episodes typical of this strategy are unlikely to have had a major impact on levels of immune activation^26^; consistent with our finding of equivalent long-term increase in CD4 cell counts in the groups. There was a small numerical excess of death, serious AIDS and non-AIDS events in the PI-mono group that was not statistically significant. In many cases other risk factors for these serious clinical events were present so a direct aetiological role of viral rebound (or associated immune activation) is unlikely. The exception is the three participants who had evidence of HIV encephalopathy in the PI-mono group that are likely to represent a specific risk of virological escape due to poor CNS penetration in a few individuals (not a generalised CNS risk of PI monotherapy, given that we found no evidence of neurocognitive impairment with PI monotherapy in systematic testing performed as part of the main trial, or in a CSF sub-study).^27^^,^^28^ Overall, we cannot exclude the possibility of a small excess risk of HIV disease-related events and this potential risk should be weighed when considering use of this strategy.

Our trial design and the novel primary outcome we used—loss of future treatment options**—**was selected to be the most relevant to evaluating the intervention tested in the trial: a strategic management approach using PI monotherapy, rather than a trial evaluating the virological efficacy of PI monotherapy as a regimen *per se.* As highlighted above, recommendations in treatment guidelines are strongly influenced by the primary efficacy endpoint used in most HIV drug trials, namely viral load suppression, following advice from regulatory agencies for licensing studies.^29^^,^^30^ However, in the context of a strategy, this outcome is uninformative. Our primary, resistance-based outcome parameter may be of more relevance, and should be considered, for future pragmatic trials that investigate ART treatment strategies for long-term care. More publicly-funded strategic trials which adopt outcomes that are more relevant to patients and clinicians are needed.^31^ Treatment guidelines are often the gatekeeper to determine which interventions are selected by clinicians or, in a centralised healthcare system, made available by the funders for selection by clinicians. However, the fact that many participants (and their clinicians) in PIVOT elected to continue PI monotherapy following the dissemination of the results from the main trial attests to the more sophisticated decision-making framework of clinical practice, influenced but not always constrained by clinical practice guidelines.

The potential limitations of this analysis are that entry to the second stage required re-consent with a risk of bias introduced by non-random attrition. However, the high retention rate (90%) and similarity of baseline characteristics between those who continued and the full trial population, suggests any such bias is likely to have been small. Similarly, the overall return rate of report forms remained high during follow-up and balanced between groups. Thus, the results during the second stage are likely to remain fundamentally a comparison between two randomised groups. Treatment was open-label, which was unavoidable in a strategy trial with selection of individual drugs left to the clinician and participant. However, this pragmatic design is a strength rather than a limitation of the trial, increasing generalisability. Bias in assessing and reporting virological outcomes is unlikely given the standardised protocol-mandated management in the first stage; and in the second stage where the similar frequency of viral load testing in the two groups, approximately 6-monthly, is consistent with standard practice for stable patients and suggests that routine care was followed in both groups. There is a possibility that resistance testing may not have been as fastidious as in the first stage of the trial, although it is unlikely that this could have changed the findings markedly. The trial was done entirely within the UK health system and the findings are unlikely to be generalisable to resource-limited settings where regular monitoring of viral load may not be available.

In summary, our results confirm the high potency and high genetic barrier to resistance of the PI drug class. This permits creative, drug-sparing strategies that may provide a broader range of approaches to enable individualised, optimised, patient-centred care; and that may appeal to patients. In selected people, such as those experiencing NRTI toxicity or who prefer to be managed without NRTIs entirely, this may include PI monotherapy, but this should be reserved for people who have a prolonged period of prior viral suppression and a high CD4 count nadir,^23^ and who do not have established or underlying risks for HIV-related brain disease; and for settings where there is access to frequent viral load monitoring (especially in the first year after switch) to enable prompt reintroduction of NRTI(s) when needed, which is an essential element of this approach. The possibility of a small excess risk of serious clinical events needs to be weighed in the treatment decision.

## Contributors

NP and DD designed the study. AA-P, AC, IW, MJ, CO, FC, VL, AW, MG and JF enrolled participants into the study. NP, WS, AA-P, KS and DD contributed to the coordination and oversight of the study. WS and DD did the statistical analysis and directly accessed and verified the underlying data. All authors participated in data interpretation. The manuscript was drafted by NP and DD. All authors provided input into the report and approved the final version of the manuscript.

## Data sharing statement

Anonymised individual participant data and study documents can be requested from the corresponding author (Nicholas Paton) and will be made available, subject to approval of the Trial Steering Committee, from 6 months after publication of this paper.

## Declaration of interests

NP reports grants to institution from Janssen; and honoraria for lectures from Janssen. AA-P reports grants to institution from Janssen, ViiV Healthcare and Gilead Sciences and advisory board fees from ViiV Healthcare. AC reports contracts to run clinical trials with payments to institution from Gilead Sciences, ViiV Healthcare, MSD and GSK; honoraria for lectures from MSD; support for attending meetings from ViiV Healthcare and Gilead Sciences; advisory board fees from Gilead Sciences, ViiV Healthcare and Theratechnologies. CO reports grants to institution from Janssen, Gilead, ViiV Healthcare, MSD and Astrazeneca; honoraria for lectures from Janssen, Gilead, ViiV Healthcare and MSD; and unpaid appointments as President of the Medical Women's Federation and a member of the International AIDS Society governing council. MG reports advisory board fees from Gilead. All other authors declare no competing interests.

## Appendix A Supplementary data

Supplementary Appendix

PIVOT_protocol v4 0_untracked_07 June 2013
