# Supplementary material for: Long-term efficacy and safety of a treatment strategy for HIV infection using protease inhibitor monotherapy: 8-year routine clinical care follow-up from a randomised, controlled, open-label pragmatic trial (PIVOT)
Source: eClinicalMedicine. 2024 Feb 10;69:102457. doi: 10.1016/j.eclinm.2024.102457 (PMC10867418; doi:10.1016/j.eclinm.2024.102457)
Supplement: Supplementary Appendix [file mmc1.docx]

**SUPPLEMENTARY APPENDIX**

**Long-term efficacy and safety of a treatment strategy for HIV infection using protease inhibitor monotherapy: 8-year routine clinical follow-up from a randomised, controlled, open-label, pragmatic trial (PIVOT)**

**The PIVOT Trial Team are:**

**Participating UK Sites:** Elton John Centre, Brighton: Martin Fisher, Amanda Clarke, Wendy Hadley, David Stacey. Royal Free Hospital, London: Margaret Johnson, Pat Byrne. Mortimer Market Centre, London: Ian Williams, Nahum De Esteban, Pierre Pellegrino, Lewis Haddow, Alejandro Arenas-Pinto. Barts & The London Hospital: Chloe Orkin, James Hand, Carl De Souza, Lisa Murthen, Andrew Crawford-Jones. Royal Berkshire Hospital, Reading: Fabian Chen, Ruth Wilson, Elizabeth Green, John Masterson. Manchester Royal Infirmary: Vincent Lee, Kamlesh Patel, Rebecca Howe. St Mary's Hospital, London: Alan Winston, Scott Mullaney. Southmead Hospital, Bristol: Mark Gompels, Louise Jennings. Royal Liverpool University Hospital: Nicholas Beeching, Rebecca Tamaklo. Guys and St Thomas’ Hospital, London: Julie Fox, Alistair Teague, Isabelle Jendrulek, Juan Manuel Tiraboschi. North Manchester General Hospital: Ed Wilkins, Yvonne Clowes, Andrew Thompson. Central Middlesex Hospital: Gary Brook, Manoj Trivedi. Avenue House Clinic, Eastbourne: Kazeem Aderogba, Martin Jones. Gloucester Royal Hospital: Andrew DeBurgh-Thomas, Liz Jones. Homerton University Hospital, London: Iain Reeves, Sifiso Mguni. James Cook University Hospital, Middlesbrough: David Chadwick, Pauline Spence, Nellie Nkhoma. Derriford Hospital, Plymouth: Zoe Warwick, Suzanne Price, Sally Read. Royal Bournemouth Hospital: Elbushra Herieka, James Walker, Ruth Woodward. Southend University Hospital: John Day, Laura Hilton. St Mary's Hospital, Portsmouth: Veerakathy Harinda, Helen Blackman. St George's Hospital, London: Phillip Hay, Wendy Mejewska, Olanike Okolo. Royal Victoria Infirmary, Newcastle: Edmund Ong, Karen Martin, Lee Munro. Royal Hallamshire Hospital, Sheffield: David Dockrell, Lynne Smart. North Middlesex University Hospital: Jonathan Ainsworth, Anele Waters. Queen Elizabeth Hospital, Woolwich: Stephen Kegg, Sara McNamara. Birmingham Heartlands Hospital: Steve Taylor, Gerry Gilleran. Chelsea & Westminster Hospital, London: Brian Gazzard, Jane Rowlands. University Hospital of Coventry: Sris Allan, Rumun Sandhu. Ealing Hospital, London: Nigel O’Farrell, Sheena Quaid. Harrogate District Hospital: Fabiola Martin, Caroline Bennett. Northwick Park Hospital: Moses Kapembwa. St James’ Hospital, Leeds: Jane Minton, James Calderwood. King's College Hospital, London: Frank Post, Lucy Campbell, Emily Wandolo. Leicester Royal Infirmary: Adrian Palfreeman, Linda Mashonganyika. Luton & Dunstable Hospital: Thambiah Balachandran, Memory Kakowa. Newham University Hospital, London: Rebecca O’Connell, Cheryl Tanawa. Edith Cavell Hospital, Peterborough: Sinna Jebakumar, Lesley Hagger. Royal Victoria Hospital, Belfast: Say Quah, Sinead McKernan. York Teaching Hospital: Charles Lacey, Sarah Douglas, Sarah Russell-Sharpe, Christine Brewer. Western General Hospital, Edinburgh: Clifford Leen, Sheila Morris. Barking Hospital, London: Sharmin Obeyesekera, Shirley Williams. Norfolk and Norwich University Hospital: Nelson David. Worcester Royal Hospital: Mark Roberts, Julie Wollaston.

**MRC Clinical Trials Unit at UCL:** Nicholas Paton, Wolfgang Stöhr, Alejandro Arenas-Pinto, Karen Scott, David Dunn, Emma Beaumont, Sue Fleck, Mark Hall, Susie Hennings, Ischa Kummeling, Sara Martins, Ellen Owen-Powell, Karen Sanders, Fionna van Hooff, Livia Vivas, Ellen White.

**Independent event reviewer**: Brian Angus

**Trial Steering Committee:** Andrew Freedman (Chair), Ben Cromerty, Danielle Mercey, Sarah Fidler, Estee Torok, Abdel Babiker, Brian Gazzard, Chloe Orkin, Nicholas Paton.

**Data Monitoring Committee**: Tim Peto (Chair), David Lalloo, Andrew Phillips and Robert James.

**Supplementary tables**

**Table 1 Case report form completion in the second phase of the trial**

| **Data collection cycle (year)** | **Case report forms returned** | | |
| --- | --- | --- | --- |
|  | **OT (n= 257)** | **PI-mono (n = 248)** | **Overall (n= 505)** |
| 2015 | 256 (100%) | 238 (96%) | 494 (98%) |
| 2016 | 248 (96%) | 228 (92%) | 476 (94%) |
| 2018 | 243 (95%) | 223 (90%) | 466 (92%) |

**Table 2. Baseline characteristics at randomisation: participants with additional follow-up**

|  | **OT group (n=257)** | **PI-mono group (n=248)** |
| --- | --- | --- |
| Drug class at entry  PI  NNRTI | 119 (46%)  138 (54%) | 114 (46%)  134 (54%) |
| Age (years) | 44 (37-49) | 44 (39-50) |
| Female | 57 (22%) | 61 (25%) |
| Route of infection  Homosexual  Heterosexual  Other | 157 (61%)  92 (36%)  8 (3%) | 147 (59%)  89 (36%)  12 (5%) |
| Ethnicity  White  Black  Other | 184 (72%)  61 (24%)  12 (5%) | 165 (67%)  72 (29%)  11 (4%) |
| Nadir CD4 count (cells per mm^3^) | 188 (90-256) | 170 (80-236) |
| Baseline CD4 count (cells per mm^3^) | 512 (385-654) | 521 (403-713) |
| Duration undetectable VL (months) | 37 (17-64) | 40 (22-66) |

Data are n (%) or median (IQR). OT=ongoing triple therapy. PI-mono=protease inhibitor monotherapy. NNRTI=non-nucleoside reverse transcriptase inhibitor

**Supplementary figures**

**Trial profile**

Randomised: n=587

Excluded: n=108

30 previous ART change due to
 unsatisfactory VL response

28 VL ≥50 copies/ml at screening or previous 24 weeks

19 did not return after screening

8 not on 2NRTI +NNRTI/PI regimen

29 had other reasons

(6 had multiple reasons)

Participants assessed for eligibility: n=695

**Allocated to Ongoing Triple Therapy:** n=291

- Received allocated therapy: n=291
- Did not receive allocated therapy: n=0

**Allocated to PI Monotherapy:** n=296

- Received allocated therapy: n=290
- Did not receive allocated therapy: n=6

4 patient decision

2 developed adverse event after switch
 from NNRTI to PI & never stopped NRTIs

**During first stage**:

Died: n = 1

Withdrawal or lost to follow-up: n=11

**During first stage**:

Died: n = 6

Withdrawal or lost to follow-up: n=5

**Transition to second stage: n=279**

Attending site that stopped after first stage: n=0

Participant did not consent: n=22

**Second stage analysis: n=257**

Died: n=1

Withdrawal or lost to follow-up: n=11

to treat analysis: n=296

**Transition to second stage: n=285**

Attending site that stopped after first stage: n=6

Participant did not consent: n=31

**Second stage analysis: n=248**

Died: n=1

Withdrawal or lost to follow-up: n=7

to treat analysis: n=296

**Time to discontinuation of PI monotherapy treatment, by initial protease inhibitor**


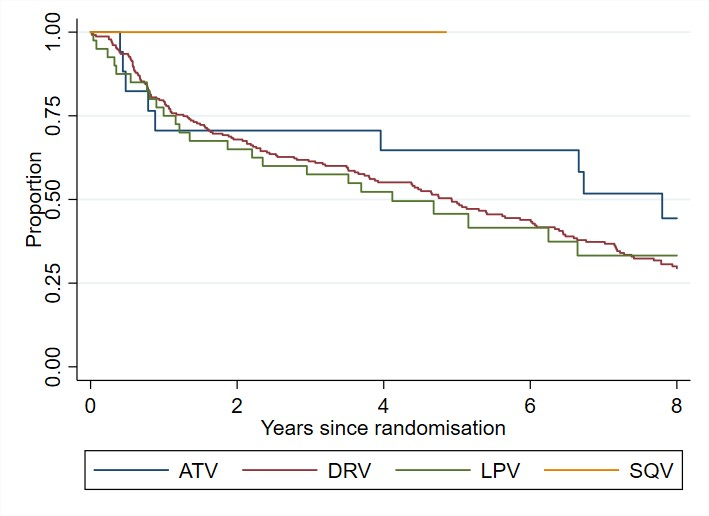


ATV, atazanavir; DRV, darunavir; LPV, lopinavir; SQV, saquinavir

**Case report form for the second phase**
